# Caries severity and socioeconomic inequalities in a nationwide setting: data from the Italian National pathfinder in 12-years children

**DOI:** 10.1038/s41598-020-72403-x

**Published:** 2020-09-24

**Authors:** Guglielmo Campus, Fabio Cocco, Laura Strohmenger, Maria Grazia Cagetti

**Affiliations:** 1 Department of Restorative, Preventive and Pediatric Dentistry, Zahnmedizinische Kliniken (ZMK), University of Bern, Freiburgstrasse 7, 3010 Bern, Switzerland; 2 Department of Surgery, Microsurgery and Medicine Sciences, School of Dentistry, University of Sassari, Viale San Pietro, 07100 Sassari, Italy; 3 Department of Biomedical, Surgical and Dental Science, University of Milan, Via Beldiletto 1, 20142 Milan, Italy

**Keywords:** Dental diseases, Public health, Epidemiology, Population screening

## Abstract

This study presents the result of the second National pathfinder conducted in Italy on children’s oral health, reporting the prevalence and severity of caries in 12-year old children and describing the caries figure related to the socioeconomic inequalities, both at individual level and macroeconomic level. The two-digit codes related to ICDAS (International Caries Detection and Assessment System) for each tooth and the gingival bleeding score were recorded at school on 7,064 children (48.97% males and 51.03% females). The Gross National Product (GNP) per capita, the Gini Index and Unemployment rate in each Italian section, parents’ educational levels, working status, smoking habit and their ethnic background were recorded. Zero-inflated-negative-binomial (ZINB) models were run, using caries-free teeth, teeth with enamel lesions, cavitated lesions and restorations as functions of socioeconomic explanatory variables, to evaluate the effects of justifiable economic factors of geographical distribution. The mean number of enamel lesions, cavitated lesions and filled per teeth were statistically significant (p < 0.01) dissimilar among the Italian section. GNP per capita, Gini Index and Unemployment rate were significantly correlated to ICDAS scores and filled teeth. Important differences in ICDAS score values remain among children from different socioeconomic backgrounds. Efforts should be made to improve awareness and knowledge regarding oral health practice and to implement preventive programs and access to dental services in Southern Italy where the disease is still unresolved.

## Introduction

Although in several European and American countries a decrease in caries figures was described, the global burden of untreated caries lesions reported in 187 countries between 1990 and 2010, makes caries a health problem yet to be solved^1, 2^. Two limitations affect data present in literature or in data bank, namely the WHO oral health data bank^3^ or the FDI data hub^4^, are the lack of methodological consensus on the assessment methods and the statistical tools to deal with the effect of the disease, estimating the latter’s prevalence.

In Italy, few epidemiological surveys were conducted^5–7^, showing an important decline of caries prevalence, which is similar to those recorded in other Western Countries where, unlike Italy, caries prevention programs at country level have been carried out and last till now. Caries decline in Italy was confirmed by the trend recorded in Sardinian twelve years old children^7^. Recent data^8, 9^ also confirmed the decline, but findings are referred to small geographical areas and different assessment methods were used for data collection.

Epidemiological data on caries are traditionally collected through the visual detection of the lesions of clean teeth by trained examiners. The actual caries figure needs a simple, evidence-based system for caries detection, able to grade caries severity, differentiating among all stages of caries lesions^10^. Several methods are described in literature like the Caries Assessment Spectrum and Treatment (CAST)^11, 12^, the Nyvad Criteria^13, 14^ and the International Caries Detection and Assessment System (ICDAS)^15, 16^ that nowadays, is the most used.

Socioeconomic inequalities are assessed using a broad spectrum of oral indicators reflecting unmet needs for both children and adults^17, 18^. Socioeconomic indicators at individual level such as occupational status, ethnicity, educational level and individual/family income, as well as macroeconomic indicators such as Employment Rate, Gross Domestic Product (GDP) and Gini coefficient may be used to explain health inequalities in children and adults^19–21^. Children from low-income households have shown a higher caries prevalence than those from high-income households^22^, as well as a greater occurrence of untreated dental caries both in children and adults was significantly associated with the Gini coefficient^19, 21^. Differences in caries prevalence among socioeconomic groups can be attributed to different factors such as sugar intake and fluoride use, oral hygiene habits and dental check-ups attendance.

In 2016, an epidemiological survey called “National pathfinder on children’s oral health in Italy” was promoted by the WHO Collaboration Centre for Epidemiology and Community Dentistry of Milan. It was the second National Survey conducted in Italy on children’s oral health. The project aimed to examine three groups of children at the age of 4, 6 and 12 years old; the examinations started in November 2016 and were completed in June 2017.

This paper aims to present the result of the second National Survey conducted in Italy on children’s oral health, reporting caries prevalence and severity in 12-year old children and describing the caries figure related to both individual socioeconomic indicators and macroeconomic indicators.

## Methods

### Population, study design sample size

In 2017 the Italian population was of 60,589,445 (29,445,741 males and 31,143,704 females); 14.4% were less than 15 years old. The Gross National Product (GNP) *per capita* of Italy in 2017 was € 28,265, the Gini index was 33% and the Unemployment rate was 11.2%^23^.

The current survey was planned and conducted as a cross-sectional study. The survey protocol was approved by the ethical committee of the University of Sassari (Italy) (AOUNIS: 29/16). All methods were performed in accordance with the Declaration of Helsinki^24^.

The children were recruited following a multistage cluster sampling procedure, covering all the sections of the Country as suggested by the Italian National Institute of Statistics (North-Western, North-Eastern, Central, Southern and Insular Italy)^23^; cities in each section and secondary school classes in each city were selected. The sample size had to be representative of each geographical section rather than the city where the examinations were carried-out.

An information leaflet describing the aim of the project was distributed to parents/guardians of the children, requesting their child’s participation in the survey. Only children with the leaflet signed by parents/guardians were enrolled. A multistage cluster sampling was performed in the five Italian sections, secondary schools were chosen at cluster level with proportional random selection of participants for each of the counties identified in each section. Children were recruited at schools using systematic cluster sampling: every class was identified as a cluster and the class list was compiled. The first cluster was randomly selected, while the others were systematically chosen at intervals of three classes. The number of subjects was approximately the same in each class (n = 20 subjects).

A sample size for each Italian section was calculated based on an assumed prevalence of dental caries (calculated using the Decayed Missed Filled Teeth) of 43%, a standard error of 0.05 and a design effect of 2.5. A total of approximately 6,000 Italian children attending the first year of secondary school was estimated for a final self-weighting sample. In total, 7,660 children were recruited and 7,064 were examined, 3,459 males and 3,605 females; 596 (7.78% of the recruited sample) were excluded: 414 children had no parents’ signed consent and 182 were not present in the classroom at the moment of the examination.

### Data collection

Data was collected by means of clinical examinations using a plain mirror (Hahnenkratt, Königsbach, Germany) and the WHO ballpoint probe (Asa-Dental, Milan, Italy) under artificial light. Caries data was recorded using the two-digit codes related to ICDAS for each tooth surface: the former, for tooth surface classification, choosing among sound, sealed, restored, crowned or missing, and the latter, for the caries stage assessment, choosing among six scores, from sound to an extensive distinct cavity with visible dentine^15^. The score 1 (first visual change in enamel) was not recorded since it required air drying for proper evaluation^25^.

Due to the high number of children to examine, the number of raters was set at four. In order to avoid the inter-cluster fluctuation attributable to inter-examiner variability, in each class examinations were carried-out by all the raters with an equal proportion of examined subjects. The team received training and inter-examiner reliability was assessed before the start of the study; sensitivity, specificity, percentage agreement and kappa statistics were recorded. Inter-examiner reliability ranged from 0.74 and 0.85 (K-Cohen) for sound, to 0.80 and 0.85 for extensive distinct cavity with visible dentine. Intra-examiner reliability ranged from 0.84 to 0.91 for sound teeth and from 0.83 to 0.89 for distinct cavity with visible dentine^10^. Gingival bleeding score, as the percentage of periodontal sites bleeding on probing, was recorded. Each tooth was gently probed with a periodontal probe at six sites (mesial, mid, and distal on both buccal and lingual surfaces); bleeding was considered positive when it was observed 20 s. after probing^26^.

The Gross National Product (GNP) per capita, the Gini Index as a measure of income inequality and the Unemployment Rate in each Italian section were recorded and used as a measure of socioeconomic inequalities. The three macroeconomic indicators for the year 2017 were considered as follows: GDP: mean Italian GDP per capita euros 31,360^27^; Gini index values: < 0.30 equity; between 0.3 and 0.4 acceptable; > 0.4–0.6 too large; > 0.6 social unrest^28^; Unemployment rate: mean in Italy 11.2%^27^.

### Data analysis

A user-friendly electronic data capture system was used through an ad hoc prepared dataset based on File Maker platform and then transferred to the server for combining all data into one data sheet in Microsoft Excel. School, date of birth, class, examination date, gender, parents’ educational levels, working status, smoking habits, ethnic background and ICDAS records per subject were anonymously collected. Ethnicity was defined as the country of birth of the parents. People born in Italy and Europe were treated as one group because they were considered similar in cultural background. Educational level, working status, smoking habits were recorded and coded using previous standardized questionnaires^29, 30^.

The frequency and severity of caries was expressed as a proportion. The tooth was considered the unit of analysis, recording the most severe ICDAS score observed in each tooth. Frequency distributions among the different ICDAS scores and means of each ICDAS score for geographical sections were calculated. Difference in proportion was calculated following the N − 1 Chi-squared test^31–33^. The One-way ANOVA test was used to evaluate differences among means. A linear regression model was built up between ICDAS scores and GNP per capita*,* Gini Index and Unemployment rate for each geographical section as a proxy variable for socioeconomic inequalities. The ICDAS scores were not normally distributed z = 17.25, p < 0.01 for caries-free teeth (ICDAS = 0), z = 15.38 p < 0.01 for enamel scores, z = 17.04 p < 0.01 for pre-cavitated lesions and z = 20.74, p < 0.01 for cavitated lesions (ICDAS 5/6); the number of filled teeth was not normal distributed as well (z = 23.48 p < 0.01).

Multilevel models that combine individual and macroeconomic indicators were used to analyse the association among caries data and socioeconomic inequalities, stratified by the five Italian areas. Two approaches were used. In the first one, Zero-Inflated-Negative-Binomial (ZINB) models were run using caries-free teeth, teeth with enamel lesions, teeth with cavitated lesions and filled teeth as functions of individual inequalities explanatory variables (gender, parents’ nationality, parents’ educational level, working status, smoking habits). The zero-inflated regression model is a mixing specification that adds extra weight to the probability of observing a zero^34^, dividing individuals into two groups: subjects not at risk, with zero counts and probability p and potential subjects at risk with probability 1 − p. The influence of covariates may move subjects from the first to the second group and the effect of the extra zero component in the ZINB model is estimated by a logit regression. The results of the ZINB model with the covariates were related to the modelling of the extra zeroes (in the logit scale) and the negative binomial process in the natural log scale. The goodness of fit of the regression models was determined by Akaike information criterion values^35^.

In the second approach, conditional fixed-effects (Italian areas) logistic regression models^36^ were run using the previous caries figures (caries-free teeth and teeth with enamel lesion, teeth with cavitated lesions and filled teeth) as functions of macroeconomic inequalities variables: GDP, Gini index and Unemployment rate. The level of significance was set at 0.05 for all statistical analyses. The data were processed and analysed using STATA 13 software^37^.

### Human participants statement

As written in “Material and methods”, the survey protocol was approved by the ethical committee of the University of Sassari (Italy) (AOUNIS: 29/16). An information leaflet describing the aim of the project was distributed to parents/guardians of the children, requesting their child’s participation in the survey. Only children with the leaflet signed by parents/guardians were enrolled. All methods were performed in accordance with the Declaration of Helsinki (https://www.wma.net/policies-post/wma-declaration-of-helsinki-ethical-principles-for-medical-research-involving-human-subjects/).

## Results

**Figure 1:**
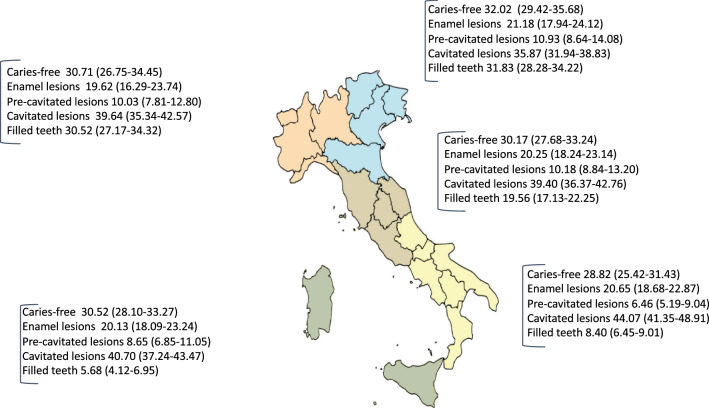
Prevalence distribution of ICDAS scores and Filled teeth (%) by Italian sections (95% CIs in parentheses); figure created with Adobe Adobe Creative Cloud by one of the author base previous publication^6^.

Overall, 30.45% (95% CI 26.95–36.43%) of the children were caries-free (ICDAS score 0), 32.05% of the females and 29.62% of the males (p = 0.08).The percentages of caries-free subjects in the different geographical sections were: 30.71% (95% CI 26.75–34.45) in North-Western, 32.02% (95% CI 29.42–35.68) in North-Eastern, 30.17% (95% CI 27.68–33.24) in Central, 28.82% (95% CI 25.42–31.43) in Southern Italy and 30.52% (95% CI 28.10–33.27) in Insular Italy (Fig. 1). No statistically significant difference in the proportion of caries-free children was observed among the Italian sections, even if the difference between children living in the South and those living in the North-East of Italy was quite high (28.82% vs 32.02% p = 0.05). Statistically significant differences in the proportion of children affected by cavitated caries lesions were observed between children living in the Southern and children living in the Northern (West/East) and Central Italian sections (p < 0.01). Children with filled teeth were statistically significantly different among sections, especially between the Northern (West/East) compared to South and Insular sections (p < 0.01).

Table 1 displays survey estimations of means and standard deviation (SD) of the number of teeth recorded with the different ICDAS scores by Italian sections adjusted for design effect. Overall, the mean number of caries-free teeth per subject was 23.93 ± 4.36 and no statistically significant differences (F = 1.87 p = 0.32) were observed among the five Italian sections. Mean enamel lesions per subject were 1.37 ± 2.28 in the whole country, while considering the different Italian sections, this data was statistically significantly different in the South of Italy 1.88 ± 1.92 compared to the North-East 1.09 ± 1.85 (F = 3.65 p < 0.01). The mean number of teeth per subject with cavitated lesions was statistically significantly different across the five geographical sections ranging from 2.60 ± 3.51 in Southern Italy to 0.62 ± 0.94 in North-East (F = 3.85 p < 0.01), with a mean of 0.85 ± 1.95 in the whole Country. Also, the mean number of filled teeth was statistically significantly different among the geographical sections (F = 3.91 p < 0.01).

**Table 1:** Mean and standard deviation of teeth per subject scored following ICDAS scores in Italy and in the five different sections.

Teeth	Italy Mean ± sd	North-West Mean ± sd	North-East Mean ± sd	Central Mean ± sd	South Mean ± sd	Insular Mean ± sd	One-way Anova F-value p-value
Caries-free	23.93 ± 4.36	24.12 ± 3.48	24.12 ± 3.89	24.08 ± 3.93	21.83 ± 5.67	24.21 ± 3.51	F = 1.87 p = 0.32
Enamel Lesions	1.37 ± 2.28	1.25 ± 2.09	1.09 ± 1.85	1.45 ± 2.22	1.88 ± 1.92	1.32 ± 2.21	F = 3.65 p < 0.01
Pre-cavitated Lesions	0.92 ± 1.22	0.91 ± 1.32	1.01 ± 1.81	1.02 ± 1.83	0.82 ± 1.00	1.04 ± 1.42	F = 1.25 p = 0.65
Cavitated lesions	0.85 ± 1.95	0.75 ± 1.18	0.62 ± 0.94	0.72 ± 1.08	2.60 ± 3.51	1.10 ± 1.62	F = 3.85 p < 0.01
Filled teeth	0.57 ± 0.85	0.82 ± 1.33	0.84 ± 1.24	0.64 ± 0.98	0.23 ± 0.71	0.22 ± 0.96	F = 3.91 p < 0.01
Number of teeth	27.71 ± 0.22	27.85 ± 0.14	27.68 ± 0.32	27.91 ± 0.08	27.36 ± 0.65	27.89 ± 0.11	–

One-way Anova was used to evaluate differences among sections.

Almost a quarter of the participants (24.25%) had gingival bleeding. The percentage of gingival sites with bleeding on probing was also statistically associated to the different geographical sections, showing 43.24% (95% CI 38.74–48.63) in the South of Italy compared to 32.16% (95% CI 28.62–34.72) in the North-West (p < 0.01) (data not in table).

**Figure 2:**
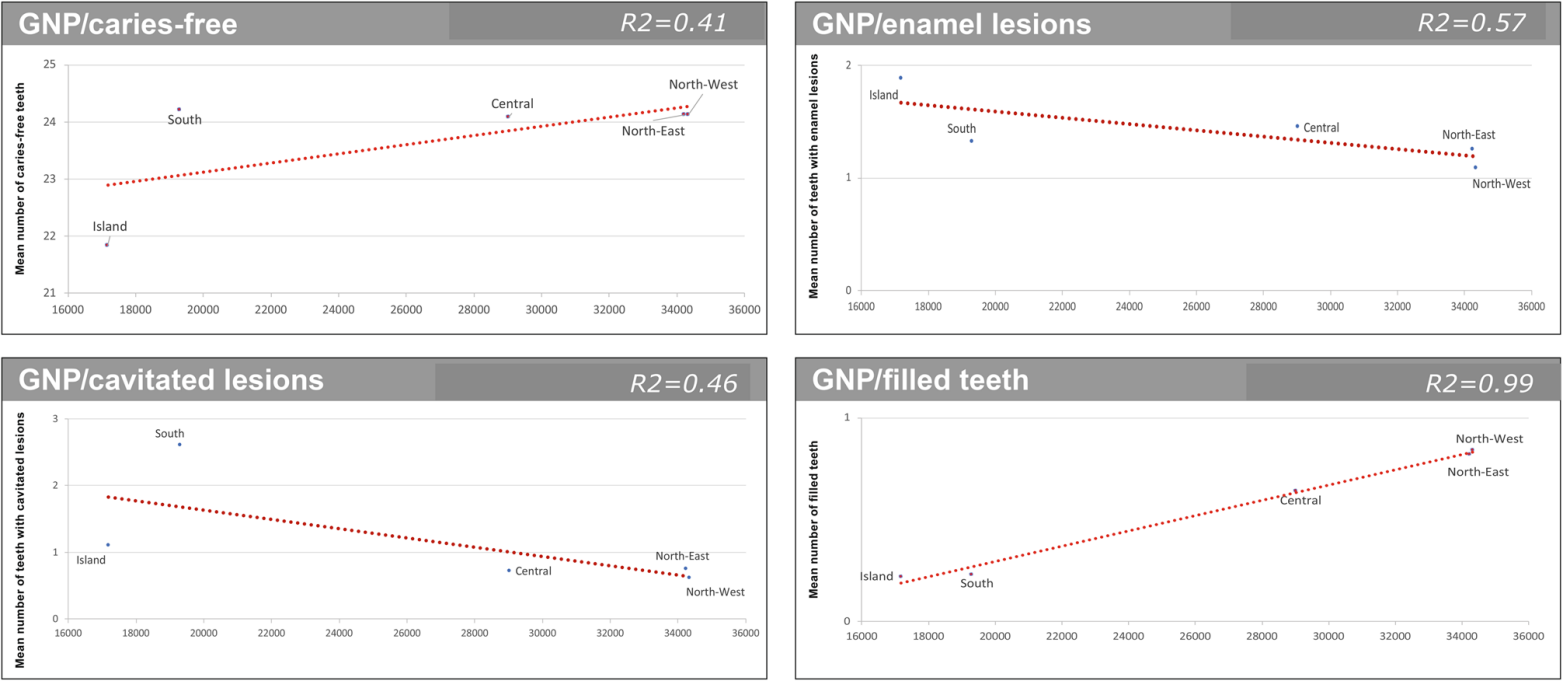
Regression analysis: caries-free teeth (ICDAS = 0), enamel lesions (ICDAS = 1/2), cavitated lesions (ICDAS5/6) and filled teeth compared to GNP *per capita* by Italian sections.

**Figure 3:**
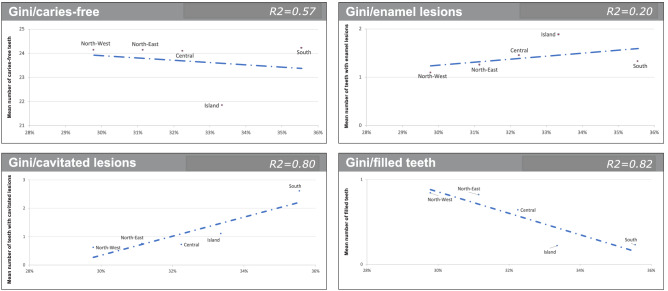
Regression analysis: caries-free teeth (ICDAS = 0), enamel lesions (ICDAS = 1/2), cavitated lesions (ICDAS5/6) and filled teeth compared to Gini Index by Italian sections.

**Figure 4:**
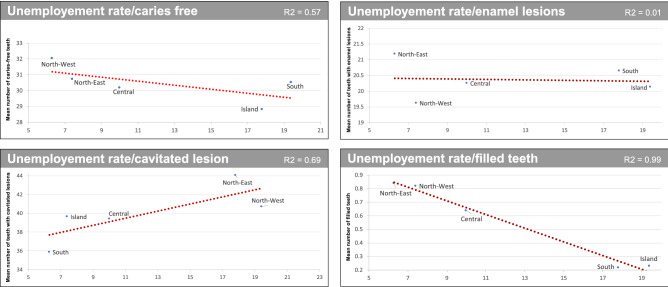
Regression analysis: caries-free teeth (ICDAS = 0), enamel lesions (ICDAS = 1/2), cavitated lesions (ICDAS5/6) and filled teeth compared to Unemployment rate by Italian sections.

GNP per capita, Gini Index and Unemployment rate were statistically significantly correlated to ICDAS scores and filled teeth (Figs. 2, 3 and 4). Regarding cavitated lesions (R^2^ = 0.46 for GNP, R^2^ = 0.80 for Gini Index and R2 = 0.69 for Unemployment rate) and regarding enamel lesions (R^2^ = 0.57 for GNP, R^2^ = 0.20 for Gini Index and R^2^ = 0.01 for Unemployment rate) the measures of socioeconomic inequalities were negative, meaning that from the bottom to the top of the socioeconomic ladder, the prevalence of cavitated lesion decreased. The opposite figure was observed for caries-free teeth (R^2^ = 0.41 for GNP and R^2^ = 0.57 for both Gini Index and Unemployment rate) and filled teeth (R^2^ = 0.99 for GNP and Unemployment rate and R^2^ = 0.82 for Gini Index).

ZINB estimates for caries levels and filled teeth stratified by Italian areas and the probability of being an extra zero by significant individual socioeconomic variables are reported in Table 2, indicating an overview of the associations between socioeconomic factors with ICDAS scores by estimated β and SE for both caries-free teeth and cavitated lesions. Caries-free teeth showed a higher probability of being an extra zero in children having a non-European father in North-West, Central, South and Insular Italy (p = 0.04), while having a non-European mother was associated to a high probability of being an extra zero in all Italian areas (p = 0.01 in North-West, North-East and Central and p < 0.01 in South and Insular Italy). The probability of being an extra zero was statistically significant higher for caries-free in subjects with fathers with a high educational level in North-West, South, Insular Italy (p = 0.04), and Central area (p = 0.03). The probability of being an extra zero was higher for cavitated caries teeth in children with non-European parents in all Italian areas or with high educational level.

**Table 2:** Zero inflated negative binomial regression analysis.

	Caries levels	North-West coeff (p-value)	North-East coeff (p-value)	Central coeff (p-value)	South coeff (p-value)	Insular coeff (p-value)
**Mother**						
High education level	Caries-free					
	Enamel lesions		− 0.51 (0.04)			
	Pre-cavitated lesions					
	Cavitated lesions	− 0.78 (< 0.01)	− 0.83 (< 0.01)	− 0.70 (0.01)	− 0.89 (< 0.01)	− 0.83 (< 0.01)
	Filled teeth	0.60 (0.01)	0.58 (0.02)	0.49 (0.04)		
Not European	Caries-free	0.84 (0.01)	1.05 (0.01)	0.71 (0.01)	0.68 (0.02)	0.72 (0.01)
	Enamel lesions	0.50 (0.04)				
	Pre-cavitated lesions					
	Cavitated lesions	− 0.94 (< 0.01)	− 0.91 (< 0.01)	− 0.94 (< 0.01)	− 0.90 (< 0.01)	− 0.94 (< 0.01)
	Filled teeth	− 0.55 (0.03)				
High working status	Caries-free					
	Enamel lesions					
	Pre-cavitated lesions					
	Cavitated lesions	− 0.52 (0.04)	− 0.50 (0.04)	− 0.54 (0.02)	− 0.53 (0.02)	− 0.64 (< 0.01)
	Filled teeth					
High smoking habits	Caries-free	− 0.71 (0.01)	− 0.61 (0.01)	− 0.56 (0.02)	0.68 (0.01)	0.70 (0.01)
	Enamel lesions	0.53 (0.03)			0.52 (0.03)	
	Pre-cavitated lesions					
	Cavitated lesions	− 0.92 (< 0.01)				
	Filled teeth					
**Father**						
High education level	Caries-free	0.49 (0.04)	0.79 (0.01)	0.51 (0.03)	0.50 (0.04)	0.64 (0.01)
	Enamel lesions	0.50 (0.04)				
	Pre-cavitated lesions	0.52 (0.03)				
	Cavitated lesions	− 0.78 (< 0.01)	− 0.66 (0.01)	− 0.74 (< 0.01)	− 0.68 (< 0.01)	− 0.80 (< 0.01)
	Filled teeth			0.51 (0.03)		0.54 (0.03)
Not European	Caries-free	0.86 (0.01)		0.87 (0.01)	0.76 (0.01)	0.78 (0.01)
	Enamel lesions		0.58 (0.02)		0.64 (0.01)	
	Pre-cavitated lesions					
	Cavitated lesions	− 0.97 (< 0.01)	− 0.84 (< 0.01)	− 0.99 (< 0.01)	− 0.81 (< 0.01)	− 0.91 (< 0.01)
	Filled teeth					
High working status	Caries-free		0.50 (0.04)			
	Enamel lesions	0.49 (0.04)				
	Pre-cavitated lesions					
	Cavitated lesions	− 0.52 (0.04)	− 0.50 (0.04)	− 0.58 (0.01)	− 0.62 (< 0.01)	− 0.64 (< 0.01)
	Filled teeth			− 0.54 (0.03)		
High smoking habits	Caries-free	0.50 (0.04)	0.63 (< 0.01)		0.51 (0.04)	0.55 (0.03)
	Enamel lesions					
	Pre-cavitated lesions					
	Cavitated lesions	0.54 (0.04)	0.62 (< 0.01)	0.60 (< 0.01)	0.73 (< 0.01)	0.70 (< 0.01)
	Filled teeth				0.52 (0.03)	

Coefficients and p-values for the Association between individual economic indicators, caries levels and filled teeth stratified by Italian Areas.

High Education level = Master degree High working status = Managers/professionals High Smoking Habits =  > 10 cigarettes/day.

A statistically significant protective effect was played by a high GDP per capita, recorded in North-West, North-East and Central (OR 0.75 95% CI 0.67–0.84) on caries-free teeth compared to a low GDP, recorded in South and Insular Italy. In North-East Italy where a Gini index of equity is reported the macroeconomic indicator had a protective role on caries compared to other Italian areas (OR = 0.69 95% CI 0.62–0.79 for caries-free and OR = 0.50 95% CI 0.46–0.84). Areas with a higher Unemployment rate as Central, South and Insular had higher risk to have cavitated lesions OR = 1.92 (95% CI 1.12–2.83) than low Unemployment rate areas as North-West and North-East (Table 3).

**Table 3:** Conditional fixed-effects regression.

	Categories	Italian areas	Caries-free OR (95% CI)	Enamel lesions OR (95% CI)	Pre-Cavitated lesions OR (95% CI	Cavitated lesions OR (95% CI)	Filled teeth OR (95% CI
GDP per capita	Low	South, Insular	1.07 (0.98–1.25)	1.28 (0.78–2.12)	2.01 (0.98–3.14)	1.48 (1.06–2.74)	0.42 (0.56–1.72)
	High	North-West, Central, North-East	0.75 (0.67–0.84)	0.75 (0.72–1.53)	0.86 (0.67–0.96)	0.54 (0.33–1.46)	1.04 (0.99–1.24)
GINI index	Equity	North-East	0.69 (0.62–0.79)	0.99 (0.81–1.09)	0.98 (0.86–1.24)	0.50 (0.46–0.84)	1.18 (0.92–1.83)
	Acceptable	North-West, Central, South, Insular	0.98 (0.92–1.76)	1.06 (0.92–1.36)	1.04 (0.78–1.33)	1.07 (0.84–1.20)	1.07 (0.94–1.34)
Unemployment rate	Low	North-West, North-East	0.81 (0.66–1.04)	0.89 (0.74–1.02)	0.78 (0.52–1.01)	0.81 (0.63–0.95)	0.88 (0.64–1.10)
	High	Central, South, Insular	1.28 (1.04–1.57)	1.41 (0.94–2.26)	1.24 (1.04–1.73)	1.92 (1.12–2.83)	1.18 (1.00–1.43)

Odds ratio (OR) and 95% CIs for the association between macro-economic indicators, caries levels and filled teeth.

## Discussion

The aim of this paper was to present the results of the second National Survey conducted in Italy on children’s oral health, reporting the prevalence and severity of caries in 12-year olds and describing the actual caries figure in relation to both individual socioeconomic indicators and macroeconomic indicators, stratified for the different geographical sections. Findings show that less than a third of the population is caries-free, with a quite strong difference between Northern and Southern Italy. Severe lesions with cavitation were more prevalent in children living in the South than in the North and Central Italy. Both macroeconomic indicators (GNP per capita, Gini Index and Unemployment rate) and individual socioeconomic indicators (nationality, educational level, working status and smoking habits) were significantly correlated to caries presence and severity and to the prevalence of restorations due to caries. Measures of economic resources, such as per capita GDP, have an important role on population health through multiple factors as welfare, higher standard of living or educational provide^19^. Worse macroeconomic indicators such as high Unemployment rate were associated with higher child mortality rates, underlying the harmful effects of the macroeconomic crises on children health^38^.

Public Health Dentistry aims to design and carry out evidence-based strategies and approaches to promote oral health and to prevent the more prevalent oral diseases, especially in frail groups like children and adolescents with low-income. In addition, scientific based guidelines on preventive strategies and methods need to be regularly provided to the medical and dental personnel, in order to react to the changes of the disease severity and distribution within a population. To achieve these objectives, it is necessary to have updated epidemiological data. In 2015 untreated caries in permanent dentition remained the most common health condition globally^39^.

The Italian Public Health System has been under scrutiny and in transition for a long time with a significant reduction of the fraction of Gross National Product spent on healthcare, on oral health care in particular. Healthcare in Italy is provided with a mixed Public and Private System, but oral healthcare is largely provided by private practitioners and it is mainly financed by direct payment by the patient or, to a lesser extent, through private insurance schemes. In this situation, the role played by socioeconomic factors on oral health outcomes is significant since they reflect at individual level knowledge and spending possibilities of each, but also at the population level, contextualizing the reality in which families live. Data of the present survey show how important is the role played by low socioeconomic indicators attributable to the father (head of the family) for the oral health of the children. Unlike what has been found in younger children where the role of the mother was fundamental for children oral health^40^, for adolescents, the role of the father is predominant since, in Italy as in other countries, the socioeconomic level of the family largely depends on him.

The previous National pathfinder was published in 2007^6^ reporting a medium–low caries prevalence in 12 years old Italian children; caries disease was recorded using the traditional WHO-DMFT index. The DMFT index, created in 1938, does not reflect the real situation of the caries figure, since different stages of the caries process are not differentiated, making it impossible to plan and adopt effective strategies for the disease control. In the actual survey, caries prevalence and severity were recorded using the ICDAS. At the best of authors’ knowledge, the present survey is the first National pathfinder carried-out using ICDAS as epidemiological caries index, able to identify the different grades of the caries process; on the other hand, the collected data are difficult to compare to those previously recorded using other diseases indices in other Countries.

Even if an unequal skewed distribution of caries figures and severity is presented in several European Countries^41^, Italian results are not really comparable to data collected in Northern European countries such as Germany^42^, Sweden^43^, the Netherlands and Norway^22, 44^, showing a quite lower caries prevalence in dentine. On the contrary, Southern European countries like France^39^ and Spain^45^ reported a dentinal caries prevalence similar to that recorded in Italy. These differences in caries figure might be explained with the different access to oral care provided by Public Oral Health Services for children and adolescents in each country.

Significant differences in caries figure distribution were noted among geographical sections. In particular, Southern Italy showed both the highest prevalence of cavitated lesions and the second-last prevalence of filled teeth. On the contrary, both Northern sections showed the highest prevalence of filled teeth with the lowest values of severe caries lesions. This disease pattern reflects the socioeconomic context in which the families live in each section as shown analysing the data in light of the socioeconomic indicators considered. People in the South of Italy, where the GNP per capita and the Unemployment rate are significantly lower and the income inequality higher than in other parts of the Country, have less access to dental care and consequently, they show a higher level of disease with a higher unmet dental need.

Bleeding on probing is an important indicator of the gingival health and it is strongly related to oral hygiene habits. In children and adolescents, the presence of gingival bleeding and calculus have been associated to sociodemographic conditions^46^. In the present survey, higher bleeding scores were recorded in children living in the South of Italy compared to those from the Northern sections. The utilization of dental services was found to be significantly associated with better gingival health in children^47^. The lack of adequate oral hygiene habits, the higher consumption of cariogenic foods and the non-regular dental check-ups might be the main reasons associated to the poor oral heath recorded in children living in low income and high inequality areas of the Country.

Dental caries and socioeconomic inequalities are under scrutiny several times^2, 19, 30^ in different age and population groups^9, 18, 47^ but the discriminating factors leading and linked to different stages of the disease are still unclear. In toddlers and kindergarten children, socioeconomic factors are found to be associated with the inequalities in caries distribution^48^. It remains to be seen whether the decline of caries in Italy will reach a plateau, as has been found in other countries^48^.

In conclusion, important differences in ICDAS scores values remain between children from different socioeconomic backgrounds. Efforts should be made to improve awareness and knowledge regarding oral health practice, to implement preventive programs and access to dental services especially in Southern Italy where the disease remains unresolved.

## Data Availability

The datasets generated during and/or analysed during the current study are available via Springer Nature’s Research Data Support service, accessible on 10.6084/m9.figshare.12090939.
